# Beyond Numerical Hessians: Higher-Order Derivatives for Machine Learning Interatomic Potentials via Automatic Differentiation

**DOI:** 10.1021/acs.jctc.4c01790

**Published:** 2025-04-25

**Authors:** Nils Gönnheimer, Karsten Reuter, Johannes T. Margraf

**Affiliations:** † Bavarian Center for Battery Technology (BayBatt), University of Bayreuth, Bayreuth 95448, Germany; ‡ Fritz Haber Institute of the Max Planck Society, Berlin 14195, Germany

## Abstract

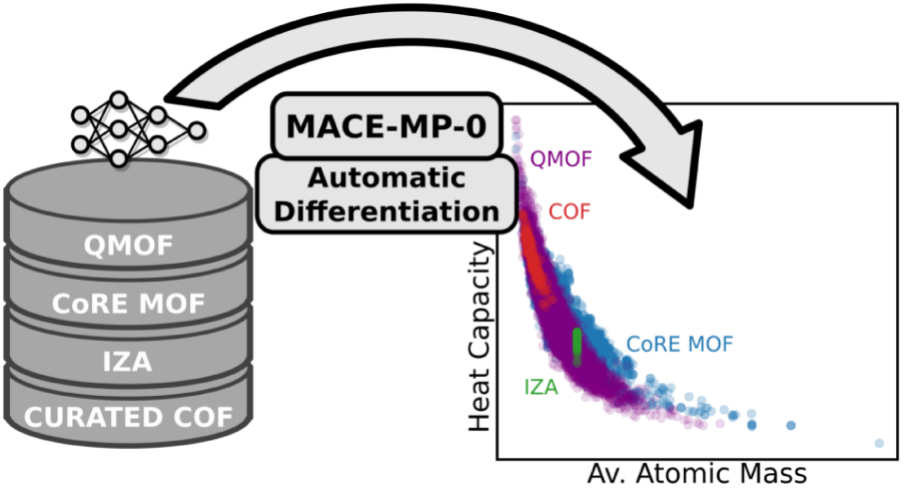

The development of machine learning interatomic potentials (MLIPs) has revolutionized computational chemistry by enhancing the accuracy of empirical force fields while retaining a large computational speed-up compared to first-principles calculations. Despite these advancements, the calculation of Hessian matrices for large systems remains challenging, in particular because analytical second-order derivatives are often not implemented. This necessitates the use of computationally expensive finite-difference methods, which can furthermore display low precision in some cases. Automatic differentiation (AD) offers a promising alternative to reduce this computational effort and makes the calculation of Hessian matrices more efficient and accurate. Here, we present the implementation of AD-based second-order derivatives for the popular MACE equivariant graph neural network architecture. The benefits of this method are showcased via a high-throughput prediction of heat capacities of porous materials with the MACE-MP-0 foundation model. This is essential for precisely describing gas adsorption in these systems and was previously possible only with bespoke ML models or expensive first-principles calculations. We find that the availability of foundation models and accurate analytical Hessian matrices offers comparable accuracy to bespoke ML models in a zero-shot manner and additionally allows for the investigation of finite-size and rounding errors in the first-principles data.

## Introduction

Quantum mechanical methods, like density functional theory (DFT), are essential in materials science and chemistry, as they allow the prediction of a wide range of properties from first-principles calculations. Unfortunately, these predictions can be computationally very demanding for complex systems or in high-throughput settings.^1−3^ This has sparked the development of machine learning interatomic potentials (MLIPs), which offer fast and accurate potential energy surface (PES) approximations, allowing the prediction of the structure, energetics, and dynamics of chemical systems.^4−8^ A major breakthrough for these methods was the introduction of accurate representations through local (atom-centered) *many-body descriptors*. These avoid the full-dimensional representation of the PES using Cartesian or internal coordinates and allow for the construction of highly accurate and scalable MLIPs. Popular examples of this approach include high-dimensional neural network potentials (HDNNPs) and Gaussian approximation potentials (GAPs).^4−6,9^

More recently, graph neural network (GNN) models that use message passing have further advanced the accuracy and transferability of MLIPs. While early GNN-based approaches, such as DTNN,^10^ SchNet,^11^ and PhysNet,^12^ were already on par with fully local descriptor-based methods, they were found to suffer from the same limitations in terms of providing an incomplete representation of the atomic environment.^13^ The atomic cluster expansion (ACE) addresses this problem by enabling the construction of local descriptors with high body orders, using complete polynomial basis functions with a linear scaling construction cost per basis function.^14−16^ Similarly, GNNs using higher-order or equivariant messages can provide a more complete representation and achieve high accuracy and transferability.^17−22^ Among these, the MACE architecture, which combines equivariance with high body order, has been found to be highly accurate in a wide variety of situations.^23^

One of the key innovations of GNN models is that they use embeddings to encode elemental species. As a consequence, GNNs can be scaled to training sets containing an arbitrary number of elemental species. Using the MPtrj dataset, which consists of snapshots from DFT relaxations of the crystals contained in the Materials Project (MP),^24^ has allowed the development of the so-called universal or foundation models like M3GNet,^25^ CHGNet,^26^ and MACE-MP-0.^27^ The latter has been found to be remarkably transferable to diverse chemical systems across the periodic table.

While all mentioned MLIPs allow fast and (given sufficient training) accurate calculations of energies and gradients (forces), some use cases also require higher-order derivatives. For example, describing thermodynamic properties, vibrational frequencies, or elastic properties (within the harmonic approximation) requires the calculation of second-order derivatives of the energy with respect to the Cartesian coordinates (i.e., the Hessian matrix). While the calculation of the forces scales linearly with the number of atoms, the Hessian scales quadratically, making its calculation for large systems a computational bottleneck.

Historically, energy derivatives of classical interatomic potentials are usually obtained analytically. For MLIPs, an analytical formulation of the derivatives is often not available; in particular, this is true for second-order derivatives. Indeed, in many cases, even the forces are not hand-implemented; instead, automatic differentiation (AD) is used.^28^ In modern machine learning frameworks like PyTorch or JAX, AD allows users to obtain derivatives of analytical quality with respect to any input parameters without manually formulating the derivatives.^29^ This is achieved by back-propagating through the computational graph constructed during the forward pass through the model. Compared with numerical derivatives, this method can be more efficient while retaining analytical precision and can easily be parallelized using vectorization. In addition to forces, AD is commonly used in backpropagation to optimize model parameters and has also found its way into computational chemistry and physics. For example, in JAX-MD, AD is employed to perform large-scale molecular dynamics (MD) calculations.^30^ AD has also been successfully used to obtain stress and heat flux for MLIPs^31^ and to compute implicit derivatives that improve the parametrization of MLIPs.^32^ AD implementations of second-order derivatives are not widely available for MLIPs. This is likely because such implementations require a second pass through the computational graph, significantly increasing computational and memory demands. Instead, Hessians are typically calculated numerically by using finite differences. Indeed, this is also a common practice in DFT calculations for solids. However, numerical derivatives are less precise than analytical derivatives and can be computationally expensive depending on the differentiation scheme.

In the first part of this article, we demonstrate the implementation of fast and accurate AD second-order derivatives for MLIPs with the MACE architecture. We compare the accuracy and performance of this method to a numerical central finite-difference implementation. In the second part, we apply this implementation to validate the heat capacities of porous materials predicted with the MACE-MP-0 foundation model relative to the DFT reference calculations. To this end, a dataset consisting of metal–organic frameworks (MOFs), covalent organic frameworks (COFs), and zeolites is used, which was previously reported by Moosavi et al. for the development of a bespoke ML model for the prediction of heat capacities.^33^ The MACE-MP-0 AD Hessians are then used for the high-throughput screening of porous materials. Beyond massively increasing the computational efficiency of the screening compared to first-principles calculations, the presented implementation also allows us to check the limitations of the DFT calculations, e.g., with respect to the step size in numerical phonon calculations or finite-size effects.

## Theory

### The Hessian Matrix

The Hessian matrix is a square matrix composed of the second-order partial derivatives of a function around a given point. It provides a compact representation of a function’s local curvature. The Hessian matrix can be formulated as
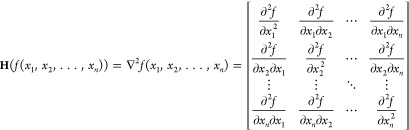
1

Here, each element  of the Hessian matrix represents the second-order partial derivative of the function *f*(*x*_1_, *x*_2_, ..., *x*_*n*_) with respect to the variables *x*_*i*_ and *x*_*j*_.

In computational chemistry and solid-state physics, calculating the Hessian matrix is crucial for understanding the PES around critical points and predicting vibrational frequencies, reaction pathways, the stability of molecular structures, and a wide range of thermodynamic properties (within the harmonic approximation).^34−36^ Here, the Hessian matrix is composed of the second-order partial derivatives of the energy *E*, with respect to the atomic positions. The (negative) first-order derivatives of the energy are the forces
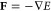
2acting on atoms. As a consequence, the Hessian matrix can be obtained by differentiating these forces. Specifically, the Hessian matrix **H** is the negative gradient of the force vector:
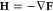
3

However, obtaining the analytical expressions for these second-order derivatives can be tedious for complex neural network architectures or electronic structure methods (in the latter case, especially because the Hellmann–Feynman theorem only holds for the first-order derivatives). Therefore, the central finite difference method is often employed to approximate the elements of the Hessian matrix for both MLIPs and (periodic) DFT calculations. This numerical technique involves displacing a single component *x*_*i*_ of the atomic position vector by a small step size *h* in both positive and negative directions. For a single element of the Hessian matrix, the approximation is then given by
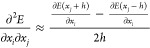
4

Here, the precision of the numerical derivatives depends on the judicious selection of *h*, and the calculation of the Hessian matrix is computationally demanding because two force evaluations are required for each component of each atomic position vector, yielding six force evaluations per atom.

### Automatic Differentiation

**Figure 1:**
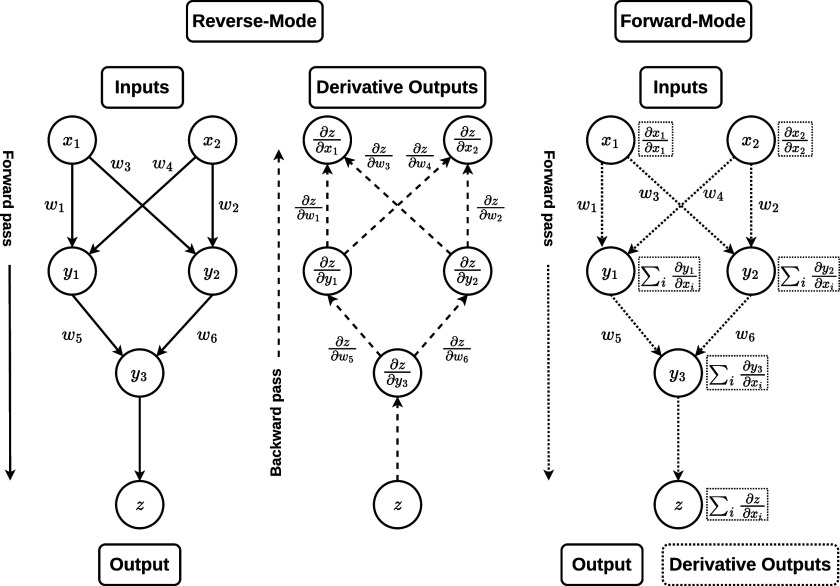
Comparison between reverse-mode and forward-mode AD. The reverse mode involves the construction of a computational graph (left), followed by backward propagation to compute derivatives (middle). In contrast, the forward mode AD (right) simultaneously constructs the graph and performs a forward propagation.^28,29^ The figure is adapted from Baydin et al.^28^ (CC-BY 4.0).

Instead of relying on numerical differentiation, machine learning tools such as PyTorch and JAX offer the option of using AD to calculate derivatives. In many MLIPs, such as MACE, forces are already calculated using AD. There are two different modes of AD: forward mode and reverse mode; see Figure 1. In both modes, a computational graph of the function is constructed, consisting of nodes (variables, parameters, intermediate values, constants, and operations within the computation) and edges (the flow of data from one operation to another and dependencies between variables and operations in the computation).^28,29^

In forward-mode AD, derivatives are calculated simultaneously with the construction of the graph. In reverse-mode AD, derivatives are obtained through a backward pass through the graph using the chain rule. Both modes result in fast derivatives of analytical quality. By combination of both modes, it is possible to efficiently compute second-order derivatives. The order and method of applying these modes are interchangeable, ensuring that the Hessian matrix is correctly obtained in any case.

**Figure 2:**
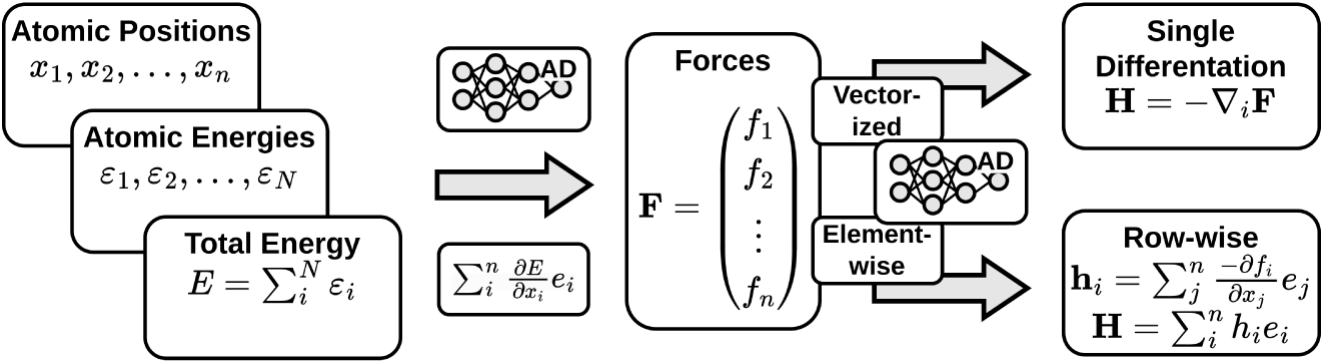
Schematic workflow for obtaining the Hessian matrix using MACE. Starting from the atomic positions, atomic energies, and total energy, the forces are derived from the total energy using AD. To obtain the Hessian matrix, two options are available: vectorized differentiation or element-wise differentiation using a second iteration of AD.

To calculate the Hessian matrix in the MACE architecture, we perform a backward propagation through the already existing computational graph, which is constructed during the AD step used for force calculation. Specifically, in the existing PyTorch implementation of the MACE potential, native reverse-mode AD is already used to backpropagate from the energies to the forces. By tracking these computations in the form of a computational graph, a second backward pass can be used to obtain a Hessian matrix. This minimizes unnecessary computational overhead, which would be caused by calculating the Hessian matrix from scratch using a combination of forward and reverse AD, since it exploits the computations already performed for the force calculation. In practice, there are two ways to implement this: in the vectorized approach, the full force vector (containing all force components for all atoms) is passed through the computational graph, and as an output, the complete Hessian matrix is obtained. In the element-wise approach, each force component is passed through the computational graph separately to obtain a single row of the Hessian matrix. While the vectorized approach can outperform the element-wise approach for small systems, its fast-growing memory demand limits its applicability. In our implementation, both variants are available and dynamically chosen according to the system size. This leads to additional computational gains for small systems. The schematic workflow of how the Hessian matrix is obtained is shown in Figure 2.

### Microscopic Heat Capacity

In the harmonic approximation, the PES around the equilibrium geometry of a system is approximated by a quadratic form, simplifying the calculation of vibrational frequencies. Mathematically, this can be expressed as
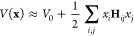
5where *V*(**x**) is the potential energy and *V*_0_ is the potential energy at the equilibrium position *x*_0_. Various thermodynamic properties can be computed by using the Hessian matrix. To do this, it is necessary to diagonalize the mass-weighted Hessian matrix (**F**^(*m*)^)
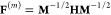
6where **M** is the diagonal matrix of the atomic masses, and to solve its eigenvalue problem to determine the vibrational frequencies. This can be formally written as
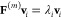
7where λ_*i*_ are the eigenvalues corresponding to the squared vibrational frequencies, and **v**_*i*_ are the eigenvectors representing the vibrational modes. The angular frequency ω_*i*_ for each mode is given by . By converting the angular frequencies, using
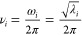
8we derive the vibrational frequency ν_*i*_. These vibrational frequencies can then be used for constructing vibrational spectra (e.g., IR or Raman spectra) or different thermodynamic quantities like the heat capacity *C*.

The heat capacity is the energy required to raise the temperature of a substance by one Kelvin. The macroscopic formulation of heat capacity involves the amount of heat *Q* required to change the temperature of a system by a certain amount. There are two main types of heat capacities: the heat capacity at constant volume (*C*_V_)
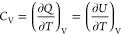
9

where *T* is the temperature, *U* is the internal energy of the system, and the heat capacity at constant pressure (*C*_P_)
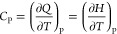
10where *H* is the enthalpy of the system. The relation between *C*_P_ and *C*_V_ for an ideal gas can be derived from the first law of thermodynamics and is given by
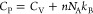
11where *n* is the number of moles of the gas, *N*_A_ is the Avogadro constant, and *k*_B_ is the Boltzmann constant. To obtain the specific heat capacity, the heat capacity is divided by the molecular mass (*M*):
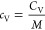
12

Describing the heat capacity within a microscopic picture, the energy required to raise the temperature is determined by the vibrations of the atoms within the system, which can be seen as the heat capacity of a phonon gas with these distinct vibrational modes.^37^ Here, the Planck distribution gives the occupation of each vibrational mode with frequency ν_*i*_ at a given temperature *T*. The heat capacity is then obtained by summing the contributions from all vibrational modes
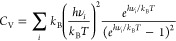
13where *h* is the Planck constant. This formulation of heat capacity represents a generalization of the Einstein model for solids, which, instead of assuming the same frequency for all modes, uses the frequencies obtained from the real Hessian matrix.^38^ According to eq 13, at low temperatures, the heat capacity approaches zero because high-frequency vibrational modes are not occupied. As the temperature increases, more vibrational modes become excited, leading to an increase in heat capacity. Eventually, at high temperatures, the heat capacity converges to a constant value of 3*R*, where *R* is the ideal gas constant, because all vibrational modes are fully active.^33,38^

## Methods

All calculations were performed using the pretrained MACE-MP-0 foundation model with the ‘medium’ model size and double-precision (float64) arithmetic unless otherwise stated. The studies presented here are based on a dataset of porous materials reported in ref (33), where the authors employed the Perdew–Burke–Ernzerhof (PBE)^39^ exchange-correlation functional with DFT-D3(BJ)^40^ dispersion corrections in the CP2K code. The Phonopy package^41^ was used to numerically determine vibrational frequencies, with a step size of h = 0.01 Å. The dataset comprises 233 porous materials, including 215 MOFs, nine COFs, and nine zeolites. The structures were optimized by following the procedure used for the CURATED-COF database.^42^

To evaluate the accuracy of the AD implementation for the Hessian matrix against the numerical central finite difference method, 10 structures were randomly selected. Specifically, these were two COFs (12022N2 and 20560N3), three zeolites (AFR, NPT, and SAS), and five MOFs (RSM0059, RSM0122, RSM0788, RSM1440, and RSM1854). We employed two error metrics for this analysis: maximum absolute error (MaxE) and mean absolute error (MAE), averaging the results over all structures. For the performance comparison between AD and the numerical implementation, we used the MOF RSM0011 from the abovementioned dataset. This MOF was expanded into supercells ranging from 1 × 1 × 1 to 4 × 4 × 4 (including noncubic supercells), resulting in 20 different configurations, of which 16 had distinct atom counts. For each supercell, the numerical and AD Hessian matrices were computed five times, and the mean calculation times for each system size are reported. For the heat capacity comparison, we used the aforementioned DFT calculations and compared them with the MACE-MP-0 model using the D3(BJ) dispersion correction. Cell parameters for the MACE-MP-0 calculations were optimized using the Fréchet-Cell-Filter and a BFGS optimizer to a *f*_max_ of 0.005 eV/Å, starting from the DFT-optimized structures. To obtain vibrational frequencies for the calculation of heat capacities, the AD implementation of the Hessian matrix was used for MACE-MP-0 calculations, while the contribution of the dispersion correction was computed separately via numerical differentiation. All heat capacities shown in this work are at a temperature of 300 K.

To study finite-size effects, we expanded the 233 porous materials into 2 × 2 × 2 supercells and determined their heat capacities, which were then compared to those of the primitive unit cells. We used unit cells optimized with MACE-MP-0 and D3(BJ), duplicating them in each direction to obtain a supercell eight times the size. These expanded systems contain up to 1920 atoms. In the large-scale screening, the complete ML dataset from ref (33) was used, which includes over 31 000 porous materials, primarily MOFs, but also COFs and zeolites. For all materials, the cell parameters were optimized using the same procedure for heat capacity comparison as DFT, except without dispersion corrections. For 1.75% of the systems in the dataset, the determination of the heat capacity failed due to a lack of convergence in the geometry optimization or due to memory issues.

All calculations were performed using NVIDIA A100 80GB PCIe or NVIDIA A100 40GB PCIe GPUs. All reported timings were exclusively obtained on an NVIDIA A100 80GB PCIe GPU together with an Intel Xeon Platinum 8358 CPU with 32 cores. The numerical differentiation for obtaining Hessian matrices of the D3 correction^40^ was performed on 18 cores of an Intel Xeon IceLake-SP processor (Platinum 8360Y) with 72 cores.

## Results and Discussion

### Evaluation and Benchmarking

**Figure 3:**
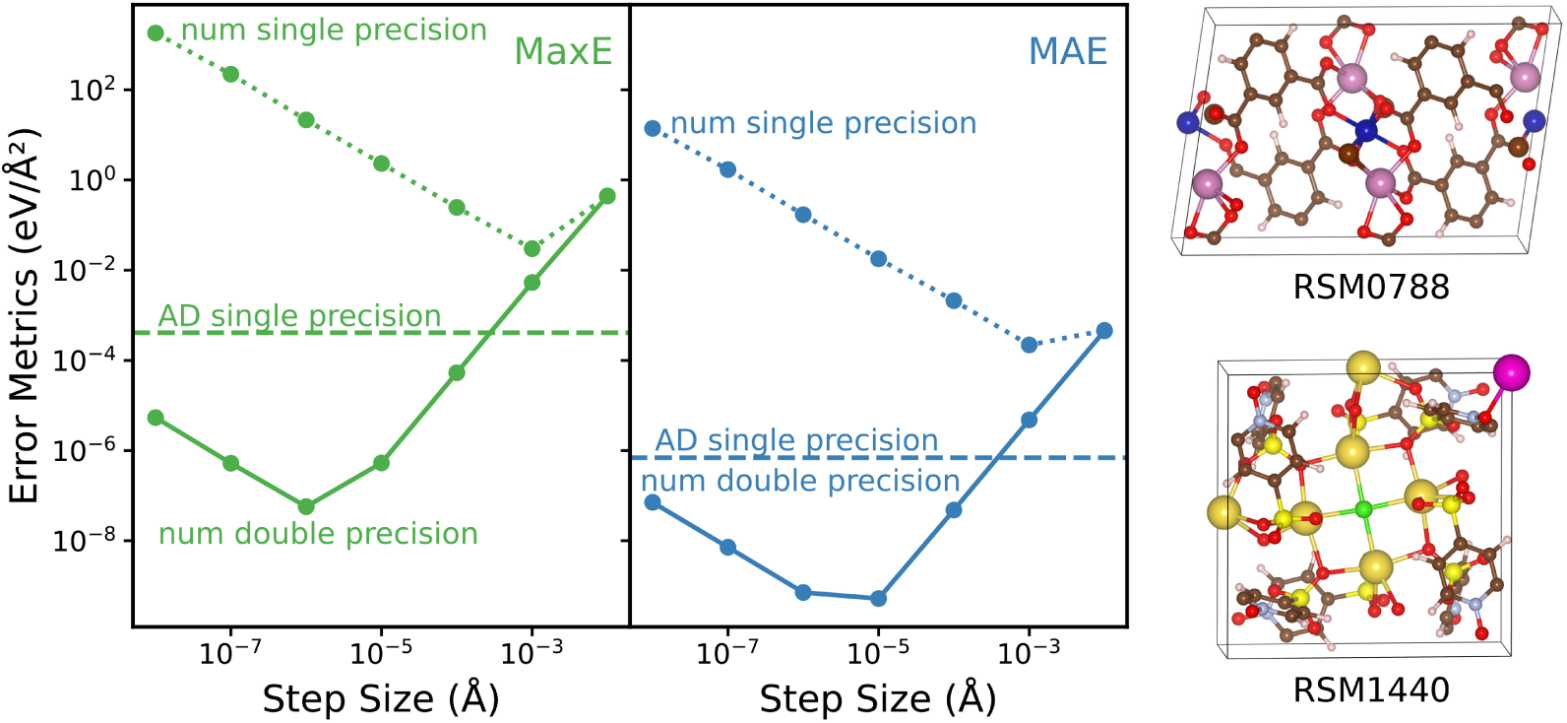
Comparison of numerical single and double-precision and AD single-precision Hessian matrix elements, relative to the double-precision AD reference. The maximum error (MaxE, green; left) and the mean absolute error (MAE, blue; center) are shown for different step sizes *h* in the numerical differentiation. The horizontal lines represent the single-precision AD Hessians. All errors are calculated relative to the double-precision AD reference and averaged for 10 different porous materials, of which two are shown on the right side.

To benchmark the numerical precision of AD Hessian matrix elements, numerical Hessians were computed with both single- and double-precision MACE models using various step sizes, *h* (see Figure 3). Here, the double-precision AD matrix elements are used as a reference. These plots display the well-known U-shape of numerical derivative errors, where the step size error dominates on the right-hand side, while rounding errors dominate on the left-hand side. This typically leads to minimal numerical errors for intermediate step sizes.

Unsurprisingly, numerical differentiation with the single-precision MACE-MP-0 model exhibits the largest deviation from the reference. Indeed, the single-precision AD Hessians are, on average, at least 2 orders of magnitude more accurate than the numerical ones, even for optimal step sizes. This is because of the large influence of rounding errors in this case, leading to a relatively large optimal step size of 10^–3^ Å. In contrast, double-precision numerical Hessians closely correspond to the double-precision AD ones (with maximum deviations below 10^–6^ eV/Å^2^), if optimal step sizes between 10^–5^ and 10^–6^ Å are used. This confirms the analytical accuracy of AD Hessians. Finally, the single-precision AD Hessians are also reasonably accurate, with average deviations below 10^–6^ eV/Å^2^ and maximum deviations below 10^–3^ eV/Å^2^. The increased precision of the AD Hessians (or of numerical Hessians with optimal step sizes) has substantial advantages in derived properties. For example, it significantly decreases the number of imaginary frequencies obtained from the diagonalized Hessian (see the Supporting Information).

Overall, this demonstrates that single-precision models introduce large errors in the Hessian matrix when numerical differentiation is used, while the difference is substantially smaller with AD. Additionally, it is worth noting that the commonly used step size of 0.01 Å in computational chemistry (e.g., in the Phonopy code) results in errors in Hessian matrix elements of up to 1.0 eV/Å^2^, regardless of model precision. This large step size is likely reasonable for DFT calculations, where the precision is limited by the finite convergence threshold of the self-consistent field procedure. For MLIPs, AD Hessians (or at least significantly smaller step sizes) should, however, be preferred.

**Figure 4:**
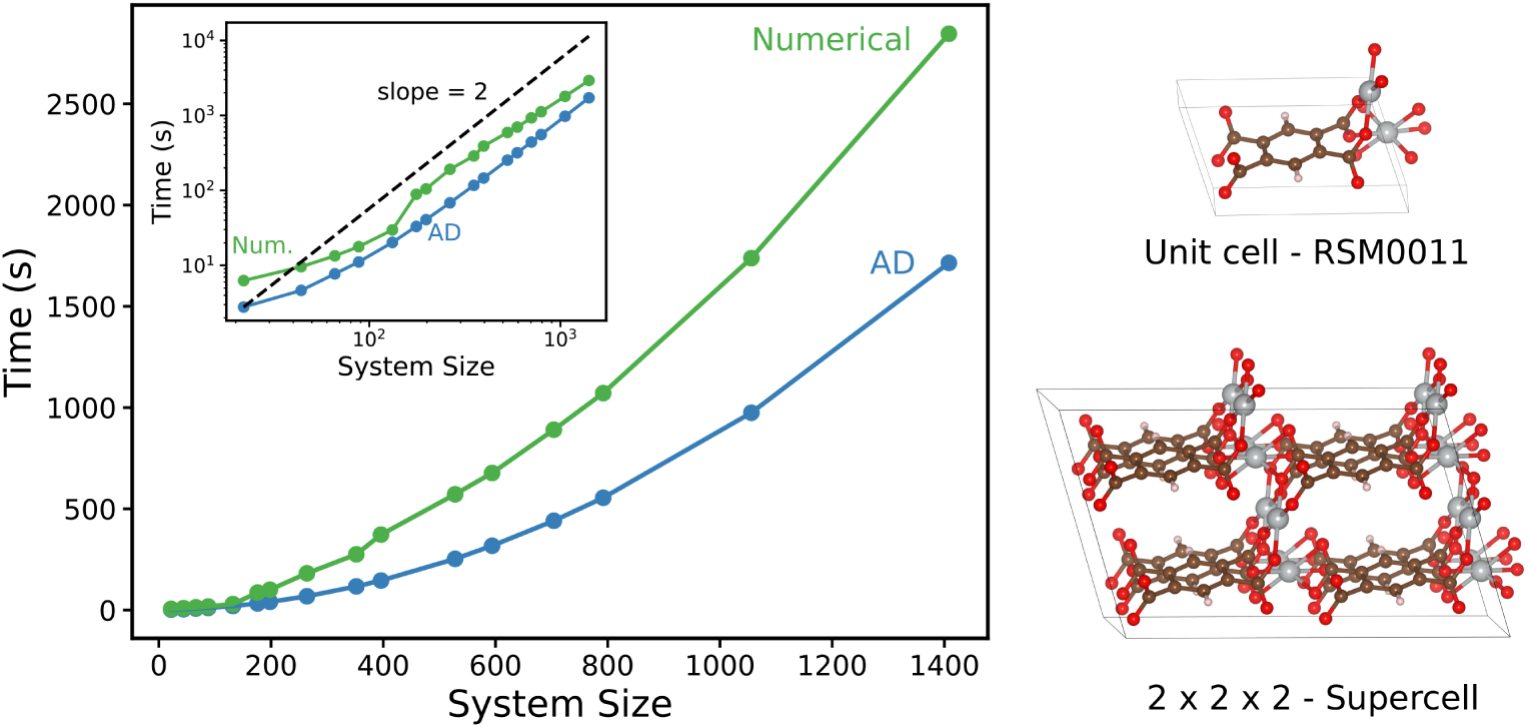
Performance comparison between numerical (green) and AD (blue) implementations for computing the Hessian matrices of differently sized supercells of the RSM0011 MOF.^33^ All calculations are performed with the pretrained double-precision MACE-MP-0 model. The inset shows the same comparison but with a double logarithmic scale and a line of slope two to show that both implementations asymptotically approach quadratic scaling. All calculations are performed with the pretrained double-precision MACE-MP-0 model.

To demonstrate the computational efficiency of the AD implementation, we compare it to numerical Hessian matrix calculations in Figure 4. This shows that the AD implementation outperforms the numerical one for all system sizes. Nevertheless, both the numerical and AD implementations scale quadratically with the size of the system. The smallest ratio between the calculation times of the numerical and AD implementations is roughly 1.5 for the 3 × 2 × 1 supercell with 132 atoms. In contrast, for medium and large systems (starting with 264 atoms), the AD implementation is more than twice as fast as the numerical one.

Since both the computational and memory costs scale quadratically with system size, the current implementation and hardware limit the calculations to periodic systems with up to ca. 1900 atoms. Note that the sparsity of the Hessian matrix could be exploited for very large systems. Due to the finite receptive field of GNN models, Hessian matrix elements between atoms that are farther apart than twice the receptive field radius (which is given by the atomic environment cutoff multiplied by the number of message-passing steps) are strictly zero. However, even for relatively local models such as MACE-MP-0, this benefit only begins to pay off for simulation cells significantly larger than 24 Å.

Additionally, we compared the AD implementation to other numerical differentiation schemes, specifically the forward- and fourth-order finite difference methods. The comparison, presented in Figures S8 and S9, shows that AD is as fast as forward finite differences—an expected result given that most MLIPs, such as MACE, are constructed from polynomials and trigonometric functions with simple derivatives. However, the AD implementation is much more accurate than the forward finite difference, particularly at commonly used step sizes. In contrast, the fourth-order method can yield precise results, approaching AD quality for a wider range of step sizes. However, it is considerably more computationally expensive.

### Application—Heat Capacities

Porous structures, such as MOFs and COFs, are ideally suited for the adsorption of small molecules. This has attracted significant attention in the context of industrial and environmental processes such as CO_2_ capture, gas separation, pollutant filtering, or gas storage.^43−45^ However, these processes are highly temperature-dependent. For example, recovering CO_2_ gas and regenerating the porous material after adsorption within a temperature swing adsorption (TSA) process requires significant amounts of energy.^33,46^ In this context, evaluating the heat capacity of these materials is crucial for assessing their suitability.

In high-throughput screening campaigns, this would imply massive computational costs at the DFT level since the heat capacity depends on the phonon frequencies (in the harmonic approximation). To address this, ref (33) reported a training set of DFT phonon calculations for porous systems and a bespoke ML model that predicts their heat capacities via an atomic decomposition approach. The fast and accurate Hessian implementation reported herein, and the recently reported MACE-MP-0 foundation model^27^ now allows a “zero-shot” approach to this problem, i.e., without explicitly training on the target property (or any MOF) at all.

**Figure 5:**
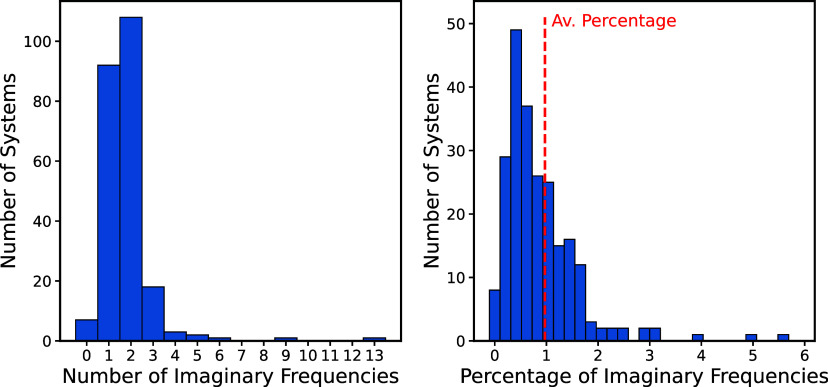
Histograms showing the distribution of the number of imaginary frequencies (left) and the percentage of imaginary frequencies (right) across various systems. The red line in the right histogram represents the average percentage of imaginary frequencies per system.

For the heat capacity calculations, imaginary frequencies were excluded, amounting to roughly 1% of the frequencies; see Figure 5. This approach aligns with the procedure chosen by Moosavi et al., where approximately 2% of the frequencies were imaginary and thus excluded. While many of these frequencies correspond to translational modes with small spuriously negative eigenvalues (as indicated by most structures having between 0 and 3 imaginary frequencies), others indicate cases where slow modes (such as those belonging to internal rotations of functional groups in linker molecules) are not fully relaxed during the geometry optimization. The comparison with the larger number of imaginary frequencies in ref (33) underscores the good numerical convergence of the optimizations used herein and the high precision of the AD Hessians.

**Figure 6:**
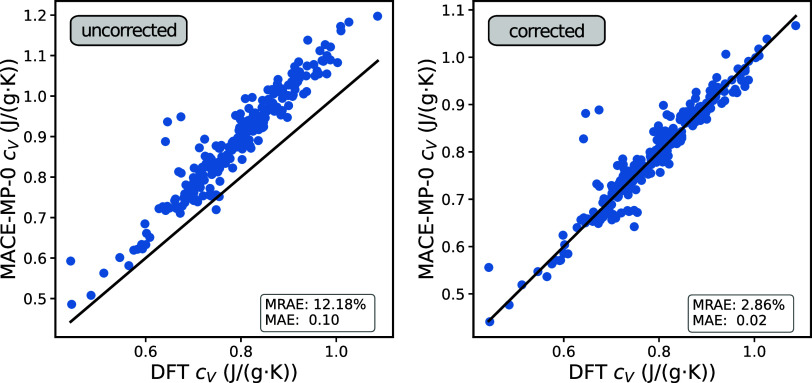
Comparison of specific heat capacities at 300 K obtained from DFT^33^ with those obtained with the uncorrected MACE-MP-0 model (left) and scaled frequencies (right).

Figure 6 shows that the MACE-MP-0 model correctly captures the overall trend of the DFT-predicted heat capacities, although a systematic overestimation is observed, especially for larger *c*_V_ values. This leads to an MAE of 0.10 J/g·K and a mean relative absolute error (MRAE) of 12.18%. Such systematic estimation errors are commonly observed in computational spectroscopy, e.g., when comparing harmonic vibrational frequencies with experiments.^47,48^ Here, it is common to apply scaling factors to the frequencies, e.g., 1.03 for harmonic frequencies obtained with the PBE functional.^49,50^ Inspired by this approach, it has also been shown that correcting the frequencies improves estimations for thermodynamic properties.^51−53^ While the motivation for frequency scaling in DFT is different (namely, in order to approximate anharmonic effects), an analogous correction can be devised herein. Indeed, the mode-softening behavior of foundation MLIPs like MACE-MP-0 appears to be quite universal and has been reported previously.^54,55^ For the 233 porous materials considered herein, this systematic underestimation of vibrational frequencies is also apparent (see Figure S7). We find that introducing a single empirical frequency scaling factor significantly reduces both MRAE and MAE. Specifically, a factor of 1.18 was employed for optimal results, reducing the MAE to 0.02 J/g·K and the MRAE to 2.86%. This is comparable to the errors obtained with a bespoke ML model in ref (33) and highlights the power of foundation models like MACE-MP-0 in zero- or few-shot contexts.

The overall quality of the MACE-MP-0 vibrational frequencies can be seen by comparing the densities of states (DOS) to the DFT reference. This reveals that the main features of the DOS are well reproduced, although a systematic underestimation of the frequencies (mode softening) is observed across all 233 porous materials (see the Supporting Information).

The overestimation of the heat capacity by the MACE-MP-0 model can be attributed to several factors. A key issue is that the model was trained on a dataset that does not include porous materials such as MOFs. Furthermore, mode softening has been observed to be a common feature of foundation models like MACE-MP-0. Beyond this, there are also differences between the computational settings used in the model’s training set (plane-wave DFT with the VASP code) and those in ref (33) (Gaussian Basis in CP2K). Heat capacities calculated through numerical derivatives are additionally subject to small but significant uncertainties, as shown in Figure 3, which illustrates the dependence on the step size (0.01 Å for the reference DFT calculations). As previously mentioned, a less accurate Hessian matrix can lead – and in this case does lead – to a higher number of imaginary frequencies in the DFT calculations, which need to be excluded. The significant role of these low-energy frequencies in determining the heat capacity is another reason for the discrepancy between the DFT and MACE-MP-0 results.

Despite these differences, the plot shows that the predicted *c*_V_ values are reasonably close to the DFT results overall, while requiring only a fraction of the time for cell optimization and Hessian matrix calculations. It is also worth emphasizing again that no retraining of the MACE-MP-0 model was needed to achieve these results. Indeed, in some respects, the MACE-MP-0 predictions can be more accurate than the DFT reference because they avoid common numerical issues in DFT phonon calculations. For example, the size of systems for which Hessian matrices can be practically calculated using DFT is limited to a few hundred atoms, even with state-of-the-art hardware and software. This limitation arises from the combination of the  DFT scaling and the quadratic scaling required for constructing the Hessian matrix. Although various methods have been developed to reduce the scaling of both processes, they all come with trade-offs.^56−58^ As a consequence, DFT heat capacities will inherently display finite-size effects. These effects can significantly impact the accuracy of thermodynamic properties.^59,60^

**Figure 7:**
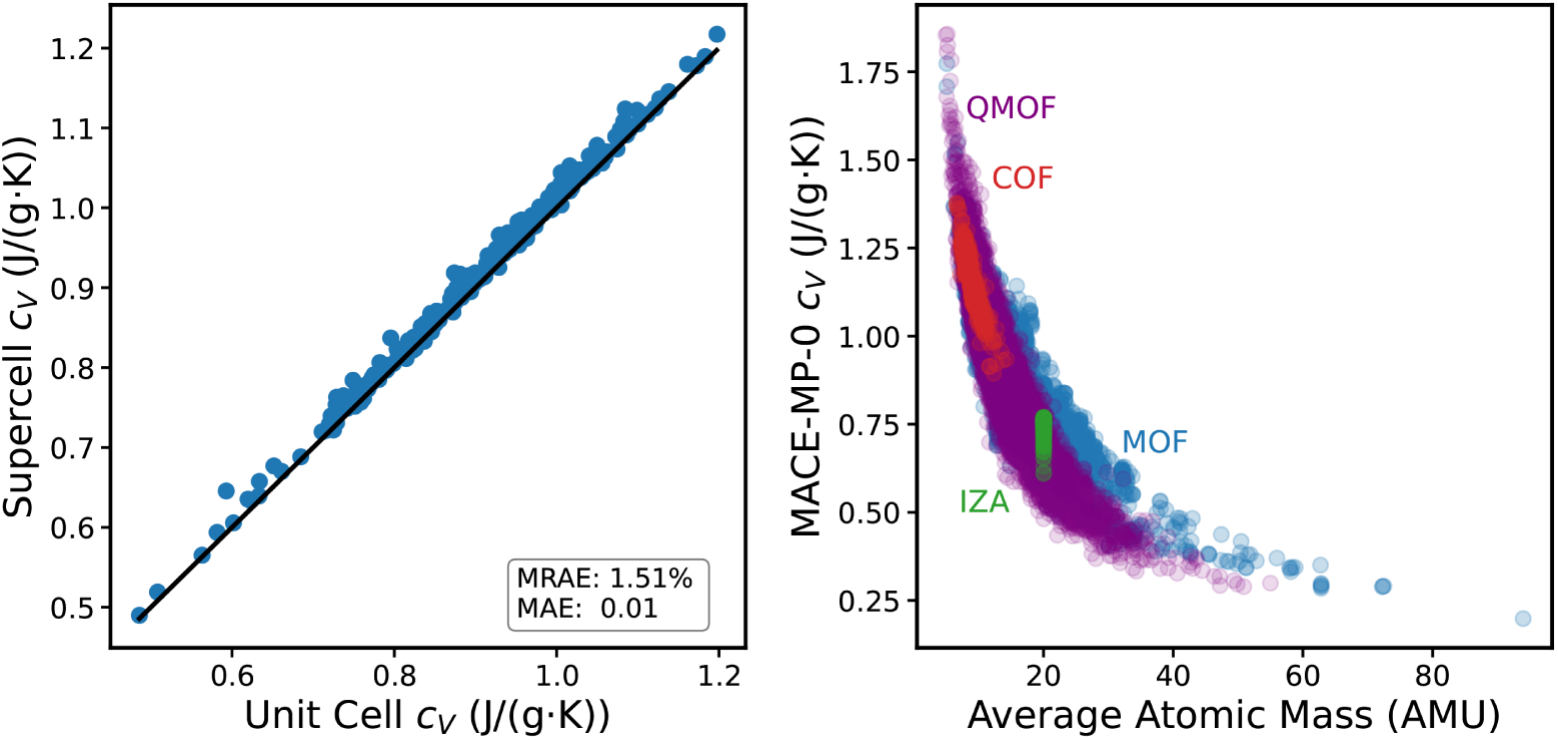
Comparison of MACE-MP-0 specific heat capacities for 1 × 1 × 1 vs 2 × 2 × 2 supercells (left) of 233 porous systems. Heat capacities at 300 K for 31 000 structures from the QMOF,^61,62^ CoRE MOF,^63^ CURATED-COF,^42^ and IZA36^64^ databases calculated with the AD implementation for MACE-MP-0 (right).

To investigate this, a comparison of heat capacities obtained from 1 × 1 × 1 and 2 × 2 × 2 supercells is shown in Figure 7 (an analysis of the number of imaginary frequencies for the supercells can be found in the Supporting Information). On average, the larger supercells display a 1.5% increase in heat capacities, which can be attributed to additional low-frequency modes in these systems. This means that finite-size effects are at least half as significant as the deviation between the scaled MACE heat capacities and the DFT reference. Monitoring the convergence of these finite-size errors would require expanding the cells further (to 3 × 3 × 3), which would result in a 27-fold increase in the number of atoms relative to the original cell. Handling such large systems (up to 6480 atoms) remains a significant challenge for the present AD Hessian implementation but could, in principle, be handled via multi-GPU parallelization. Nevertheless, even the current approach enables accurate calculation of vibrational frequencies for systems with up to 1900 atoms at near-DFT accuracy.

In addition to easily scaling to larger simulation cells, MLIPs also enable computationally efficient high-throughput screening, e.g., in materials discovery. Figure 7 (right) illustrates this by showing the heat capacities of ca. 31 000 porous materials from the QMOF,^61,62^ CoRE MOF,^63^ CURATED-COF,^42^ and IZA36^64^ databases. In agreement with ref (33), we find an inverse correlation between the heat capacity and the average atomic weight. Nonetheless, the heat capacity can vary by a factor of 2 for systems with similar average atomic mass, which strongly affects the ranking of materials for efficient CO_2_ capture. The current work provides a new tool that allows the fast and reliable estimation of heat capacities when designing novel carbon capture materials.

## Conclusions

In this work, we introduced an AD implementation for second-order derivatives in the popular MACE architecture of MLIPs. Our results show that using single-precision arithmetic and numerical differentiation (with the commonly used step size of 0.01 Å) can lead to errors as large as 1 eV/Å^2^ for individual elements of the Hessian matrix. Double-precision models are generally more accurate in this context and benefit from smaller step sizes, but they can also display significant numerical noise. In contrast, AD Hessians are both faster (by a factor of 1.5 to 2.7) and more accurate (even for single-precision models). We further demonstrated that the combination of the pretrained MACE-MP-0 model and the AD Hessian implementation is a viable alternative for predicting the heat capacities of porous materials with reasonable accuracy when compared with DFT reference data. The significant performance advantage of MACE over DFT makes it suitable for large-scale screening of thermodynamic properties, as shown for over 31 000 porous materials. Additionally, larger supercells could be investigated in order to explore the role of finite-size effects, which were found, on average, to amount to a 1.5% underestimation of the heat capacities. This deviation is on the same order of magnitude as the relative deviation between the (scaled) MACE-MP-0 and DFT heat capacities (2.9%). Importantly, all results were achieved without any fine-tuning of the pretrained MACE-MP-0 model, highlighting its ability to extrapolate to new data. Similar results were recently reported for the organic MACE-OFF23 foundation models in the context of the prediction of IR spectra.^65^ Overall, the AD Hessian implementation in MACE not only improves the accuracy and performance of Hessian matrix calculations but also offers near-DFT accuracy with significantly better scalability for larger systems and datasets. For some enhanced sampling methods, like hyperdynamics,^66^ or transition state searching methods,^67^ an on-the-fly evaluation of the lowest eigenvalue and eigenvector of the Hessian is required. These are usually approximated only in electronic structure methods because constructing the full Hessian is prohibitively expensive in this setting. With MLIPs and AD Hessians, however, it becomes feasible to compute the full Hessian on-the-fly with analytical accuracy, expanding the applicability of these methods. Importantly, this approach can easily be applied to other MLIP architectures that use AD for obtaining forces.

## Data Availability

The reference data used in this study are from ref (33) and can be accessed at 10.24435/materialscloud:p1-2y. The scripts for performing the benchmarking, DFT comparisons, and screening, and the corresponding CSV files can be found at https://github.com/Nilsgoe/AD_heat_capacity/. The AD implementation for Hessian matrices is integrated into the MACE package, available at https://github.com/ACEsuit/mace/tree/main.

## Supporting Information Available

Evaluation of the benchmarking, step size dependency of the imaginary frequencies, supercell frequency analysis, density of states, and alternative numerical differentiation approaches (PDF)
