# Supplementary material for: Beyond Numerical Hessians: Higher-Order Derivatives for Machine Learning Interatomic Potentials via Automatic Differentiation
Source: J Chem Theory Comput. 2025 Apr 25;21(9):4742–52. doi: 10.1021/acs.jctc.4c01790 (PMC12080109; doi:10.1021/acs.jctc.4c01790)
Supplement: Supplementary file 1 — ct4c01790_si_001.pdf [file ct4c01790_si_001.pdf]

Supporting Information: Beyond Numerical Hessians: Higher-Order Derivatives for Machine Learning Interatomic Potentials via Automatic Differentiation

Nils Gönnheimer,[†,‡] Karsten Reuter,[‡] and Johannes T. Margraf[*,†]

†*University of Bayreuth, Bavarian Center for Battery Technology (BayBatt), Bayreuth, 95448, Germany*

‡*Fritz Haber Institute of the Max Planck Society, Berlin, 14195, Germany*

E-mail: johannes.margraf@uni-bayreuth.de

Evaluation of the Benchmarking

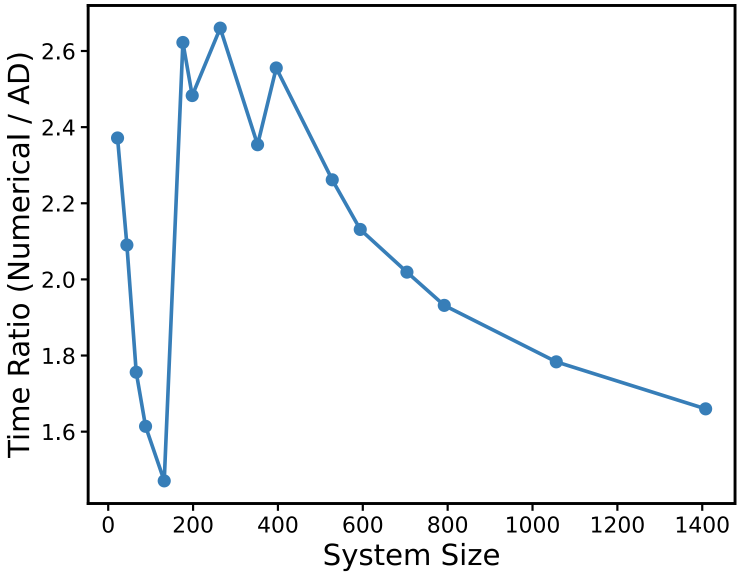

Figure S1: Ratio of the calculation time for the numerical differentiation and the AD implementation to obtain a Hessian matrix for different sized supercells of RSM0011[1].

The ratio of calculation times between numerical differentiation and the implementation of AD varies with the size of the system; see Fig. S1. For very small systems and those with between 200 and 450 atoms, the AD implementation is approximately 2.5 times faster. However, for systems with 80 to 160 atoms, the ratio drops to around 1.5. This value also appears to be the asymptotic limit for larger systems. Unfortunately, due to memory constraints, the system sizes cannot be increased further for verification.

Step Size Dependency of the Imaginary Frequencies

For the full set of 233 porous materials, we computed both the absolute number of imaginary frequencies and their percentage relative to the total number of frequencies in each system (See Fig. S2 & S3), for step sizes ranging from 0.01 to 10^{-5} Å and for the AD implementation. Here, only MACE-MP-0 and no dispersion correction has been used. Additionally, Tab. 1 shows the mean values of the two distributions. All numerical differentiation was performed using central finite differences. The comparison shows that larger step sizes produce significantly more imaginary frequencies, while smaller step sizes cause the numerical implementation to converge toward the AD implementation.

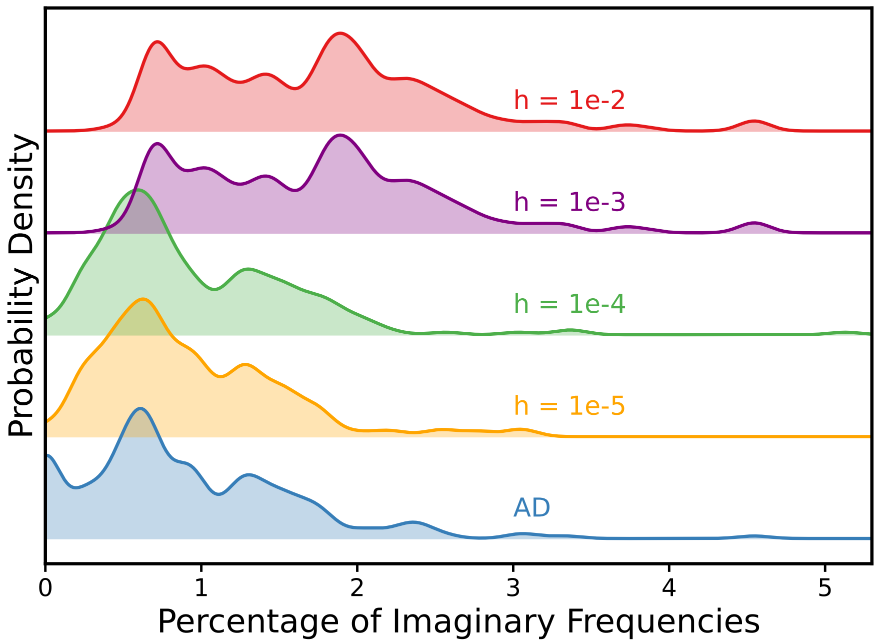

Figure S2: Comparison of the absolute number of imaginary frequencies per system obtained at various step sizes (in Å) using central finite difference versus the AD, aggregated over all 233 porous materials. Performed with the double precision MACE-MP-0 model.

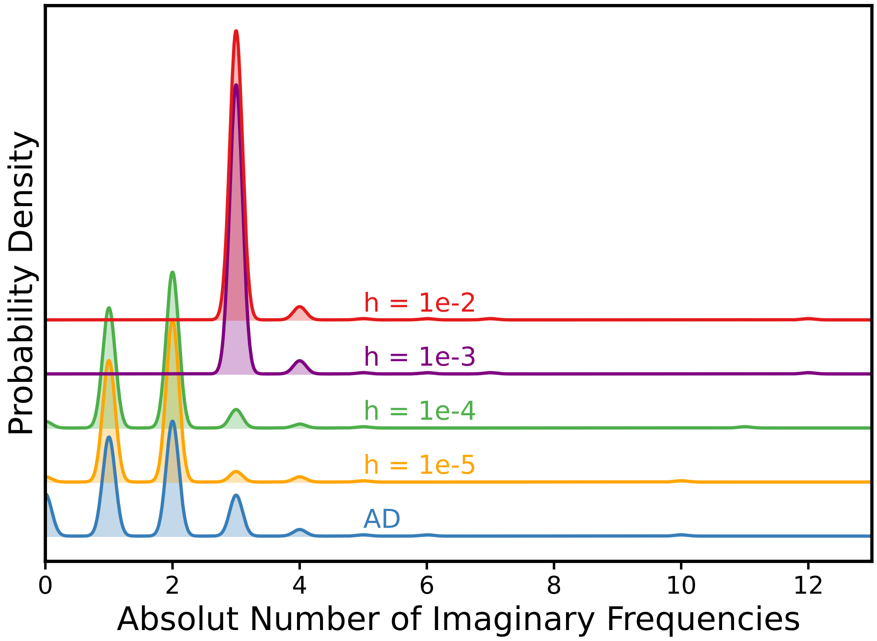

Figure S3: Comparison of the absolute number of imaginary frequencies per system obtained at various step sizes (in Å) using central finite difference versus the AD, aggregated over all 233 porous materials. Performed with the double precision MACE-MP-0 model.

Table 1: Comparison of the averaged values for percentages and absolute number of imaginary frequencies obtained at various step sizes using the central finite difference method versus the AD implementation, averaged over all 233 porous materials.

Averaged:	AD	$h = 10^{-2}$ Å	$h = 10^{-3}$ Å	$h = 10^{-4}$ Å	$h = 10^{-5}$ Å
Percentage of im. ν_i	0.89	1.74	1.74	0.94	0.92
Average number of im. ν_i	1.64	3.12	3.12	1.70	1.68

Supercell Frequency Analysis

Expanding the porous materials from unit cells to $2 \times 2 \times 2$ supercells increases the number of atoms by a factor of eight, and correspondingly, the number of vibrational modes also increases by a factor of eight, since the number of vibrational modes is $3N$ for a system with N atoms. However, the average number of vibrational modes with imaginary frequencies only doubles, increasing from approximately 1.8 to 3.5. This decreases the relative percentage of modes with imaginary frequencies from 1.0 % to 0.22 %. Some systems exhibit a disproportionately high number of vibrational modes with imaginary frequencies; see Fig. S4.

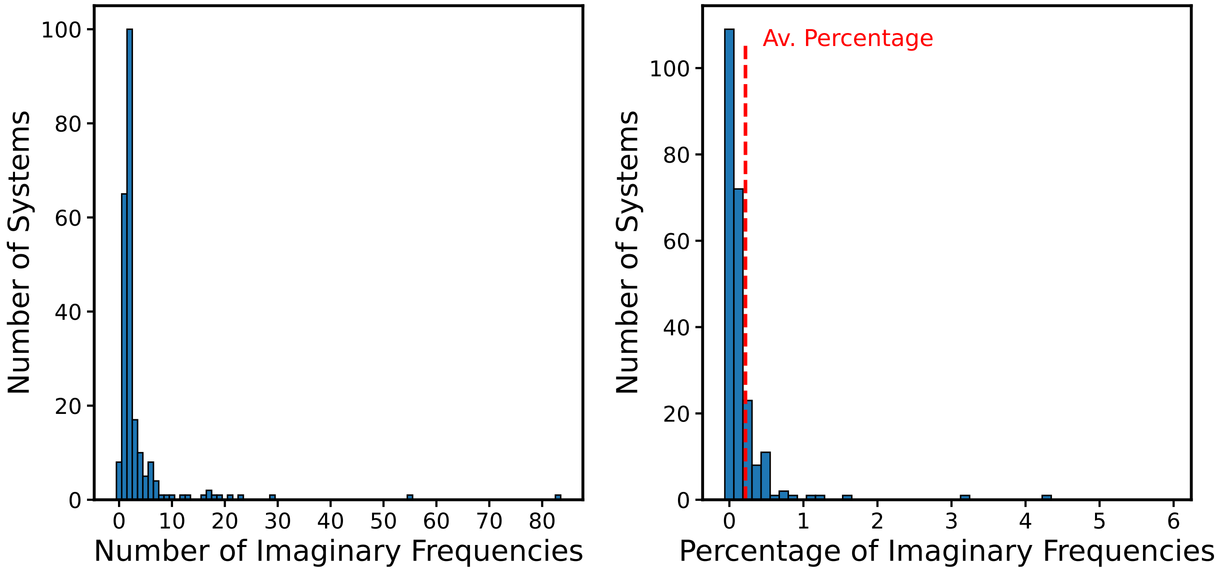

Figure S4: Histograms showing the distribution of the number of imaginary frequencies (left) and the percentage of imaginary frequencies (right) across all $2 \times 2 \times 2$ supercells. The red line in the right histogram represents the average percentage of imaginary frequencies per system.

Specifically, three systems stand out: RSM1162 with 29, RSM0210 with 55, and VFI with 83 imaginary modes. In the case of unit cells, the systems with the highest number of modes with imaginary frequencies were RWY (6), RSM0210 (9) and VFI (13). All these porous materials are shown in Fig. S5. As expected, systems with a high number of imaginary frequencies in unit cells also have a high number in $2 \times 2 \times 2$ supercells, although these values are significantly lower than eight times the original number of imaginary frequencies.

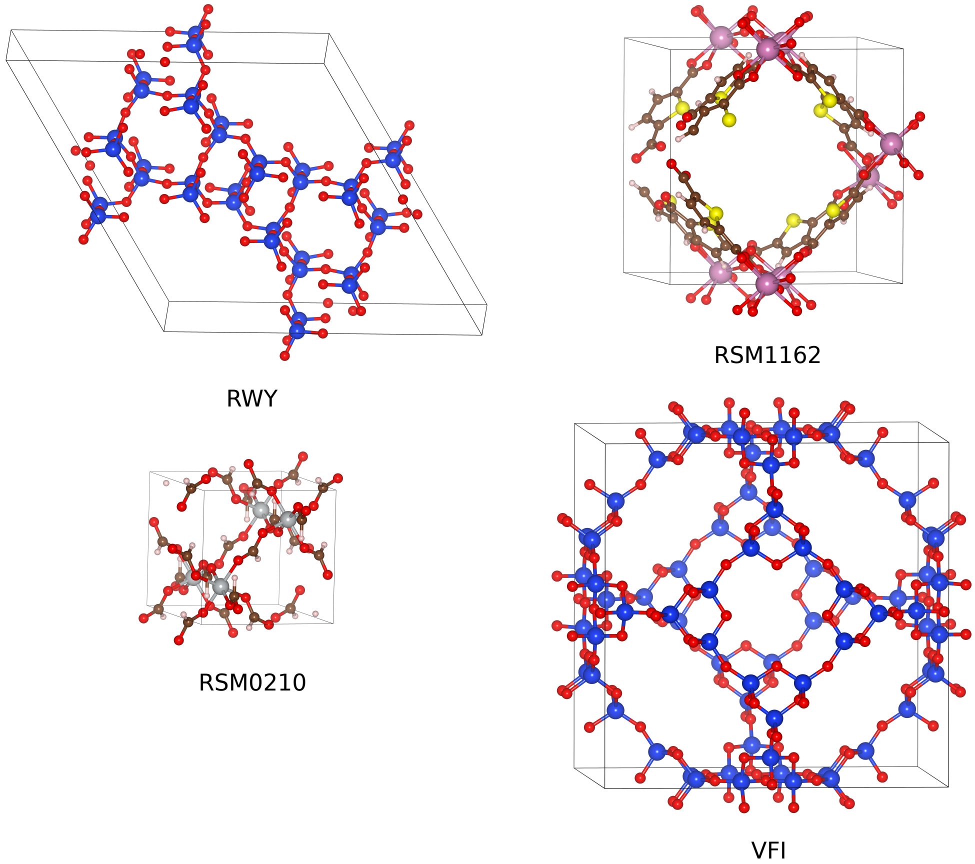

Figure S5: Unit cells of porous materials with an especially high number of vibrational modes with imaginary frequencies in rather the unit cell, the supercell, or both.

Density of States

For a deeper analysis of the MACE-MP-0 phonons, we compare the vibrational density of states (DOS) for six different porous materials (RSM0044, RSM0079, RSM0113, RSM0153, RSM0860, and RSM1208) with the DFT reference of Moosavi *et al.* in Fig. S6.

The DOS is obtained using a Gaussian broadening function:

$$g(\omega) \approx \frac{1}{\sqrt{\pi}\sigma} \sum_{s} \exp\left[-\left(\frac{\omega - \omega_s}{\sigma}\right)^2\right],$$

where σ denotes the width of the Gaussian, and ω_s represents the eigenfrequency obtained from the dynamical matrix for mode index s.

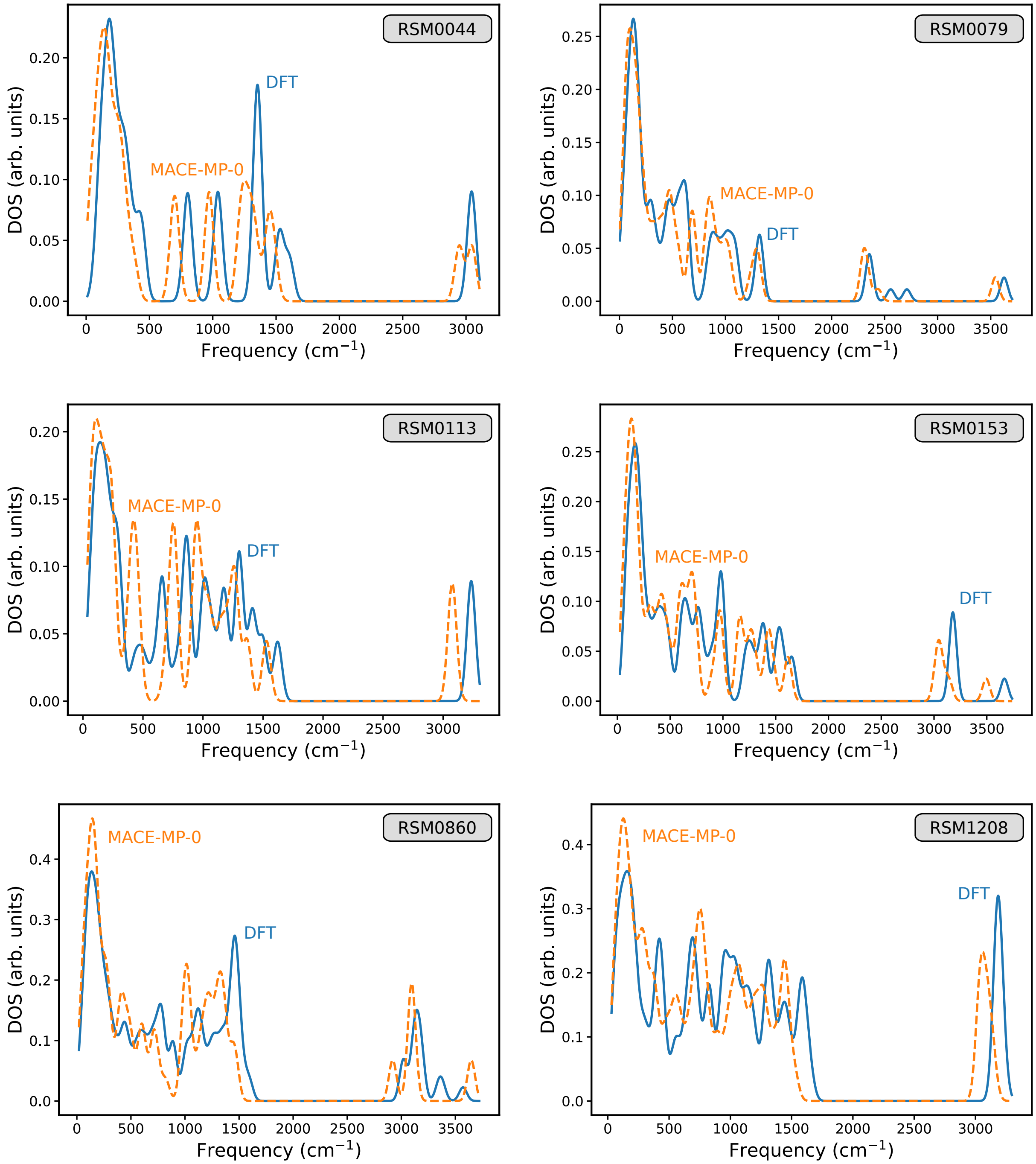

Figure S6: Vibrational density of states for six different porous materials. In orange calculated with MACE-MP-0 and the AD Hessians, in blue calculated using DFT (PBE) and phonopy, with $\sigma = 50\text{cm}^{-1}$.[1]

Additionally, we compared the frequencies of all 233 porous materials from MACE-MP-0 and DFT in Fig. S7. The MAE for all real frequencies is 80.12 cm^{-1} and the plot shows that

most frequencies are systematically underestimated by MACE-MP-0. However, there is also a small number of large imaginary frequencies for DFT that do not appear for MACE-MP-0. This is likely due to the relatively large step size used for numerical differentiation in the DFT data, as well as not fully converged DFT relaxations.

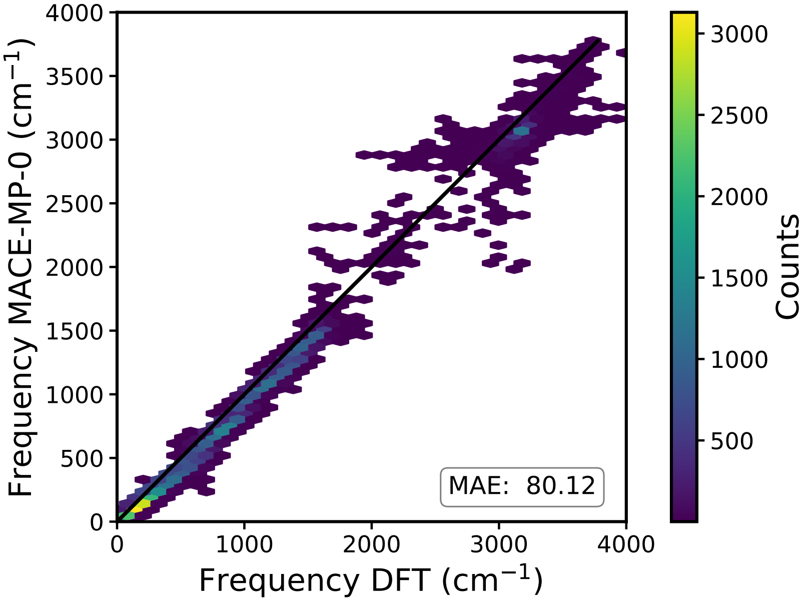

Figure S7: Comparison of the real DFT and MACE-MP-0 frequencies for all 233 porous materials by Moosavi *et al.*[1]. Imaginary frequencies have been excluded from this plot.

Alternative Numerical Differentiation Approaches

Besides the central finite difference approach discussed in the main paper, there are other methods for obtaining numerical derivatives. For comparison we also consider the faster but less precise forward numerical differentiation, and the more precise fourth-order numerical differentiation. For performance comparisons, we used supercells of RSM0011 of different sizes (see Figure S8).

The forward numerical differentiation is given by:

$$f'(x) \approx \frac{f(x+h) - f(x)}{h},$$

and the fourth-order numerical differentiation is given by:

$$f'(x) \approx \frac{-f(x+2h) + 8f(x+h) - 8f(x-h) + f(x-2h)}{12h}.$$

As expected, the fourth order method is about four times slower than the AD Hessians, while the forward scheme is of comparable computational cost to the AD implementation.

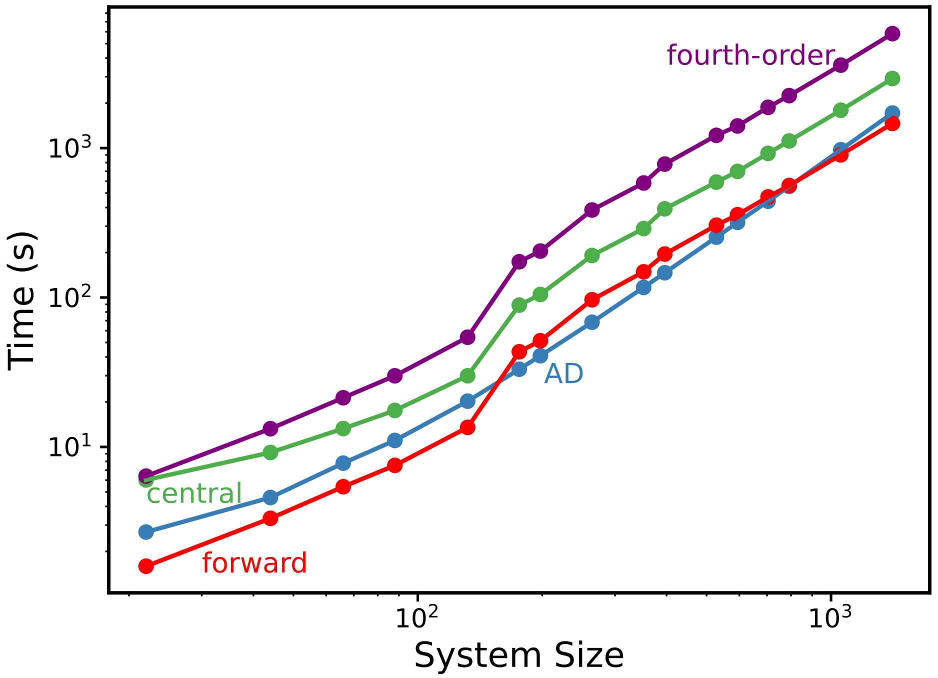

Figure S8: Computational effort comparison between three different numerical finite difference approaches (forward - red, central - green, fourth-order - purple) and the AD (blue) implementations for computing the Hessian matrices of differently sized supercells of the RSM0011 MOF[1]. All calculations are performed with the pre-trained double precision MACE-MP-0 model.

Figure S9 shows a comparison of these approaches with regard to accuracy, using the same 10 porous materials described in the main paper (two COFs (12022N2, 20560N3), three zeolites (AFR, NPT, SAS), and five MOFs (RSM0059, RSM0122, RSM0788, RSM1440, RSM1854)) and employing the same error metrics.

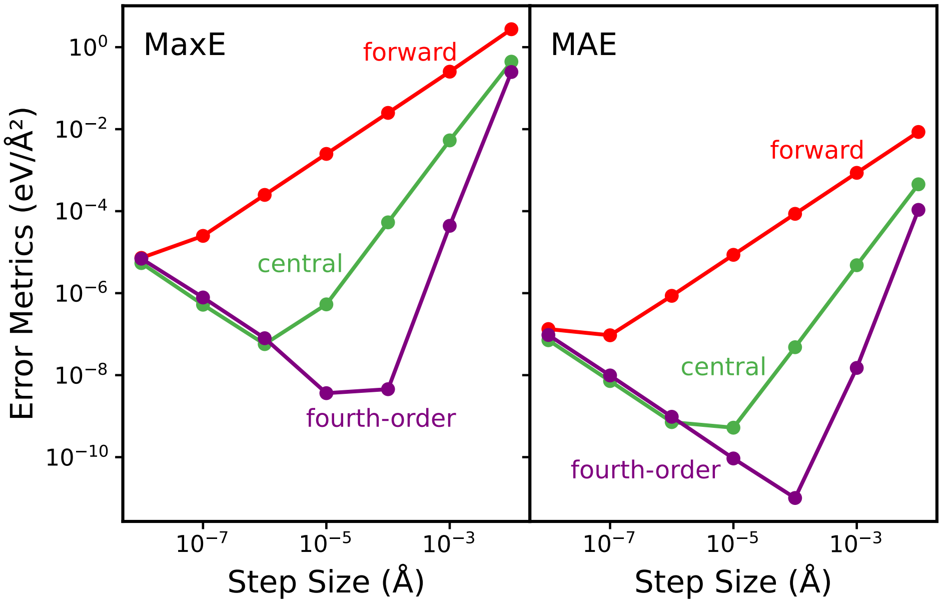

Figure S9: Comparison of the accuracy of forward (red), central (green), and fourth-order (purple) numerical differentiation for Hessian matrix elements (all performed using the double-precision MACE-MP-0 foundation model) against the double-precision AD reference. The accuracy is evaluated using two metrics: maximum error (MaxE, left) and mean absolute error (MAE, right), for different step sizes h in the numerical differentiation. Results are averaged over 10 different porous materials.

References

(1) Moosavi, S. M.; Novotny, B. A.; Ongari, D.; Moubarak, E.; Asgari, M.; Kadioglu, O.; Charalambous, C.; Ortega-Guerrero, A.; Farmahini, A. H.; Sarkisov, L.; Garcia, S.; Noé, F.; Smit, B. A data-science approach to predict the heat capacity of nanoporous materials. *Nat. Mater.* **2022**, *21*, 1419–1425.
